# Working towards a new normal: a meta-synthesis of patient-reported aspects of a good life with heart disease

**DOI:** 10.1186/s12955-025-02388-6

**Published:** 2025-06-04

**Authors:** Lisa Nebel, Timothy R. Le Butt, Christoph Herrmann-Lingen, Daniel Broschmann

**Affiliations:** 1 Department of Psychosomatic Medicine and Psychotherapy, University Medical Center Göttingen, Von-Siebold-Str. 5, Göttingen, 37075 Germany; 2 German Center for Cardiovascular Research (DZHK), Partner Site Göttingen, Robert-Koch-Str. 42a, Göttingen, 37075 Germany

**Keywords:** Heart disease, Quality of life, The good life, Normalcy, Meta-synthesis, Interpretative phenomenological analysis

## Abstract

**Aims:**

This meta-synthesis challenges the medical notion of health-related quality of life (HRQOL) by identifying aspects of the more holistic philosophical concept of the good life from the perspective of patients with heart disease.

**Design:**

Systematic meta-synthesis.

**Methods:**

Following preregistration on PROSPERO, a systematic literature search was conducted in PubMed, PsycInfo, PsycArticles, and PSYNDEX from February 2023 to March 2024. Studies focusing on the experiences of adults living with heart disease were included based on predefined criteria. Articles were assessed for methodological quality using a modified CASP tool, and data synthesis followed Interpretative Phenomenological Analysis. Reporting adheres to PRISMA and ENTREQ guidelines.

**Results:**

Forty-three articles were included, revealing the overarching theme of “Working towards a new normal,” with four sub-themes: (1) Feeling safe in my own body again, (2) Important relationships provide security and meaning, (3) Taking my life into my own hands again and (4) Living more consciously.

**Conclusions:**

While HRQOL is a valuable and widely used concept, it primarily captures quality of life from a functional perspective. Qualitative studies on individuals with heart disease reveal the additional aspects of coherence, connectedness, and self-determination that align more closely with the concept of a good life.

**Implications:**

To enhance care and counseling for patients with heart disease, the concept of HRQOL should be broadened to incorporate preferences, interests, and needs regarding life planning, aligned with the notion of a good life.

**Supplementary Information:**

The online version contains supplementary material available at 10.1186/s12955-025-02388-6.

## Introduction

In medicine, quality of life (QOL) reflects patients'subjective experiences, complementing measurable criteria of morbidity and mortality. The World Health Organization (WHO) defined QOL as an individual’s evaluation of their life circumstances against “culture and value systems […] goals, expectations, standards, and concerns” [1] (p.1403). This aligns with the philosophical concept of the Good Life, which examines how individuals shape their lives in ways they consider fulfilling – whether in terms of human flourishing [2–4], positive experiences [5], the satisfaction of desires and preferences [6], or the attainment of prudential goods [7].

However, in medical contexts, QOL often narrows to health-related aspects. Health-related QOL (HRQOL) evaluates how symptoms impact physical, psychological, and social well-being and functioning from the patient’s perspective [8]. This reflects hedonistic understandings of the Good Life, defining health as a state of pleasure [5] and illness as displeasure [5]. By addressing immediate health concerns, HRQOL overlooks broader life fulfillment shaped by values and aspirations. This reduction is particularly relevant for patients with severe conditions, which confront them with their own mortality and profoundly shape their life approach, as well as for those with chronic diseases, where experiences of illness, pain, and limitations become integral to daily life.

A prominent example of such severe and chronic conditions is heart disease, which can be congenital or acquired. It may manifest acutely, such as in myocardial infarction (MI) or cardiac arrest (CA) and also carries the risk of progressing into chronic conditions like heart failure (HF) or ischemic heart disease (IHD), which is the most diagnosed cardiac condition worldwide and the leading cause of death [9]. Improved medical care has led to a decline in mortality rates [10], resulting in more people living with severe or chronic heart disease (SCHD) [10–12].

HRQOL research has emphasized the psychosocial situation of these patients, which significantly affects somatic outcomes, such as prognosis and mortality [13, 14]. However, beyond symptom burden and functional impairment, patients who develop heart disease throughout adulthood face existential challenges. Previous meta-syntheses [15–17] and qualitative studies on survivorship [18, 19], posttraumatic growth (PTG) [20], coping [21, 22], and adjustment [23, 24], show that heart disease represents a biographical rupture: Needs for security, independence, and meaning are disrupted and significantly influence patients’ approaches to life [25]. Living with a changed body imposes limitations, profoundly altering a person’s life course [15, 16] and evoking ambivalent emotions [15, 17]. Thus, coping with the illness may present a profound challenge as it involves balancing lifestyle adjustments with affirming life [26].

While these studies recognize that illness experiences extend beyond functional health and well-being, they rarely address patients’ values, goals, and concerns essential for a fulfilling life. These aspects remain underexplored in HRQOL research, creating a gap between empirical findings and holistic care approaches. To bridge this gap, our meta-synthesis aims to enhance HRQOL research in cardiology by identifying what heart patients consider essential for a good life, using the philosophical concept of the Good Life as a theoretical framework.

## Methods

### Research question

What do patients with SCHD consider fundamental to living a good life?

### Design

This systematic review employs an interpretative meta-synthesis approach [27, 28]. To analyze patients'experiences from a first-person perspective, we utilized the well-manualized Interpretative Phenomenological Analysis (IPA) [29]. As all authors were involved in a qualitative primary study exploring patients'ideas of a good life in the context of SCHD, we engaged in supervision sessions to discuss and mitigate our assumptions throughout the analysis, adhering to the phenomenological principle of bracketing. The hermeneutic circle and the idiographic focus of IPA continuously guided our consideration of the contextual nuances present in the primary studies.

Reporting follows the Enhancing Transparency in Reporting the Synthesis of Qualitative Research (ENTREQ) guidelines [30].

### Search strategy

After registering the review on the international prospective register of systematic review protocols, PROSPERO, the study team, consisting of [author 1] and [author 2], conducted a comprehensive literature search in medical and psychological databases including PubMed, PsycInfo, PsycArticles, and PSYNDEX on 02/15/2023. The publication date was not limited. Population search terms were the Medical Subject Heading (MeSH) “Heart Diseases” in PubMed, the index term “Heart Diseases” and specific terms, such as “Angina Pectoris”, “Coronary Artery Disease”, or “Heart Arrythmias” in the psychological databases. We excluded congenital heart defects from our search, assuming that they may shape conceptions of a good life differently. Methodological search terms were “Qualitative Research (MeSH)” in PubMed, the index term “Qualitative Methods” in the psychological databases, and additional terms describing specific qualitative data collection and analysis approaches, such as “In-depth Interview*” or “Phenomenol*”. Subject search terms were “Good Life” in all databases, “QOL (MeSH)” and “Psychological Adaptation (MeSH)” in PubMed, and the index terms “QOL” and “Emotional Adjustment” in the psychological databases. These index terms include patient-centered constructs like well-being, life satisfaction, survivorship, coping, PTG, sense of coherence, and salutogenesis. Complete search strings are provided in additional file 1.

### Study selection

**Fig. 1:**
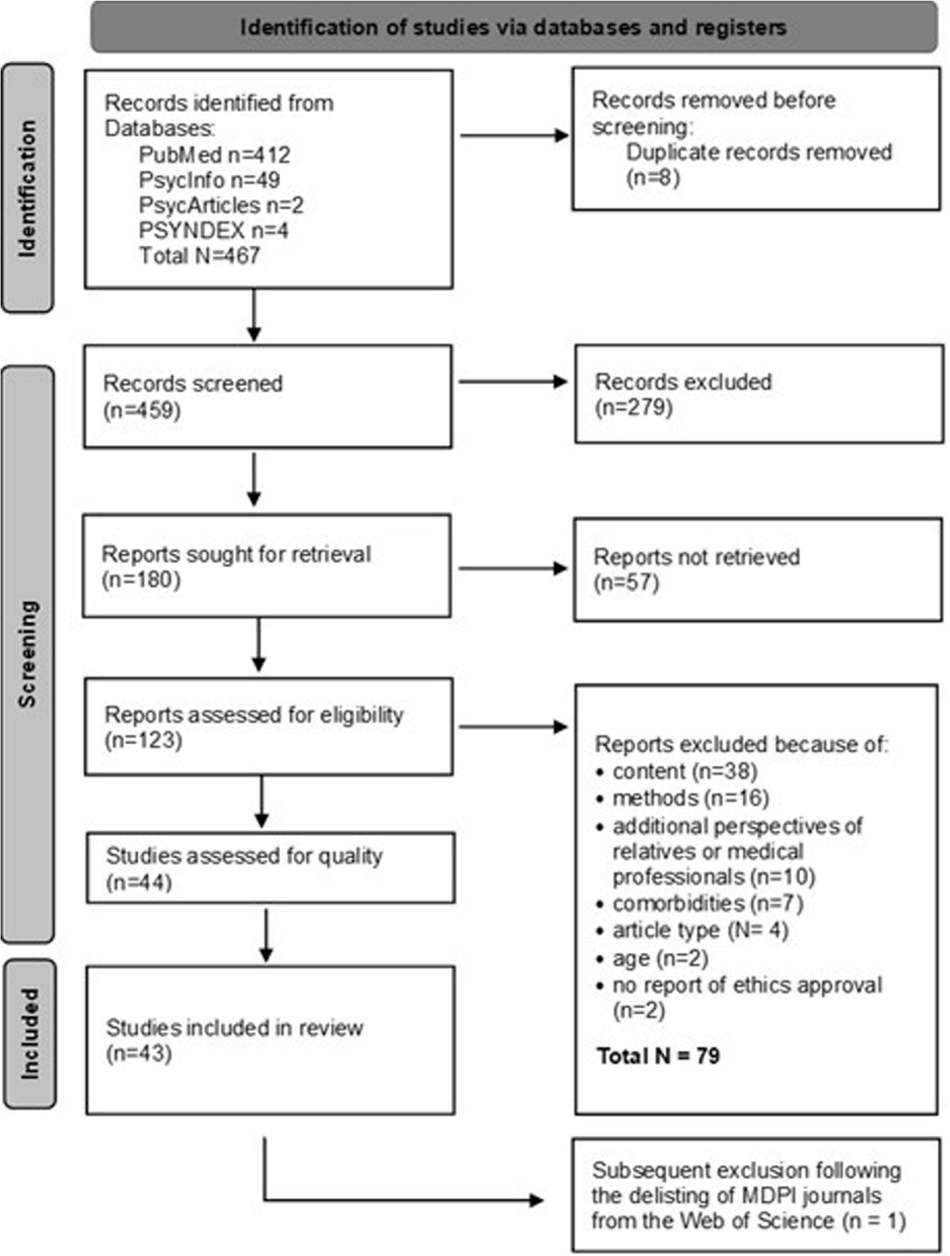
PRIMSA flow chart of the study inclusion and exclusion

Our search yielded a total of 467 articles. After removing duplicates, [author 1] and [author 2] independently screened 459 articles at the abstract level based on predefined criteria. The criteria were defined based on the PICoS-framework and included specifications regarding the population, phenomenon, and context of interest, as well as on the type of studies to be included [31]. Inclusion criteria encompassed English or German language, a qualitative study design, and a study sample involving patients aged 18 or older with SCHD. To ensure maximum variation, we included studies on various types and severities of heart diseases, including MI, CA, IHD, HF, arrhythmias, and infective heart diseases. Settings ranged from hospitalization and rehabilitation to advanced home care. Studies were excluded if they focused on congenital heart disease in individuals under 18, severe somatic comorbidities (e.g., cancer) or mental illness (e.g., major depression, schizophrenia). We also excluded studies that did not focus on the patients’ experience of heart disease, mixed-methods studies and those involving interviews with third parties to maintain a focus on qualitative data and patients'perspectives. After excluding 336 articles, we reviewed 123 full texts and included those that closely aligned with our research question, following the criterion of intensity sampling [32]. A follow-up search conducted on 02/15/2024 did not yield additional qualifying articles. The PRISMA flow chart (Fig. 1) illustrates the search process.

### Quality appraisal

In line with intensity sampling, [author 1] and [author 2] independently assessed the methodological quality of the 44 included reports. We applied a modified version of the Critical Appraisal Skills Program (CASP) tool, incorporating the Consolidated Criteria for Reporting Qualitative Research (COREQ) domain on research team and reflexivity to mitigate researcher bias [33]. Deciding criteria included COREQ reflexivity and CASP results. To differentiate between limitations in reporting and such in methodological rigor, a “somewhat” category was added to the standard response options ('yes','no','can't tell') [34]. Quality appraisal informed our synthesis through differential weighting of primary studies’ results: High-quality studies were used to develop an initial understanding of themes, moderate-quality studies to add new codes, and low-quality articles to validate the coding system without adding new codes.

### Evidence synthesis

The results section of the primary studies was defined as relevant material, categorizing direct patient quotations as first-order data, contextualizing information as second-order data, and abstract themes as third-order material. MaxQDA was used for coding, which followed the inductive process of IPA: (1) [author 1] and [author 2] independently read the articles for initial understanding. (2) While reading, we noted any linguistic or conceptual peculiarities. (3) We then developed line-by-line codes for the first high-quality article, focusing on patient quotes, contextual information, and themes. (4) By abstracting, subsuming, polarizing, enumerating or analyzing the initial codes in terms of their function and context, we developed abstract themes. (5) We repeated steps 1–4 for the other good quality articles before (6) identifying similarities and differences between themes across articles. The resulting provisional structure of themes was then used for coding moderate-quality articles. If these contained new information about the phenomenon, additional codes were generated by returning to steps 3 and 4. Finally, articles of lower quality were coded according to the established theme structure, without generating new codes. During the selection and analysis process, [author 1] and [author 2] maintained a research diary and conducted weekly briefings. [Author 4] supervised each step of the study, with [author 3] providing occasional support.

## Results

Of the 44 included articles, we identified 11 as high-quality, 16 as moderate-quality, and 17 as lower-quality based on our deciding criteria (Table 1). One lower-quality article was subsequently excluded after the publishing MDPI journal was delisted from the Web of Science in 2024 [35].

**Table 1:** Appraisal of the included studies with the CASP tool

	High-Quality Articles											Moderate-Quality Articles																Lower-Quality Articles															
**CASP/COREQ questions**	1	2	3	4	5	6	7	8	9	10	11	12	13	14	15	16	17	18	19	20	21	22	23	24	25	26	27	28	29	30	31	32	33	34	35	36	37	38	39	40	41	42	43
Clear statement of the studies aim(s)	✓	✓	✓	✓	✓	✓	✓	✓	✓	✓	✓	✓	✓	~	✓	✓	✓	✓	✓	✗	✓	✓	✓	✓	✓	✓	✓	✓	✓	✓	✓	✓	✓	✓	✓	✗	✓	✓	✓	✓	✓	✓	✓
Appropriate qualitative method	✓	✓	✓	✓	✓	✓	✓	✓	✓	✓	✓	✓	✓	✓	✓	✓	✓	✓	✓	✓	✓	✓	✓	✓	✓	✓	✓	✓	✓	✓	✓	✓	?	✓	✓	?	✓	✓	✓	✓	✓	✓	✓
Appropriate design to address the aims of research	✓	✓	✓	✓	✓	✓	✓	✓	✓	✓	✓	✓	✓	?	✓	✓	?	✓	✓	~	~	✓	✓	✓	✓	✓	✓	✓	✓	✓	✓	?	?	~	?	?	✓	✓	✓	✓	✓	?	✓
Recruitment strategy to the aims of research	✓	✓	✓	✓	?	✓	?	✓	✓	✓	~	~	✓	✓	✓	✓	~	?	?	✓	?	?	✓	✓	~	?	✓	~	?	?	✓	?	✓	?	?	?	✓	✓	?	?	?	?	~
Collection of data in a way that addressed the research issue	✓	✓	✓	✓	?	✓	?	~	?	✓	✓	✓	~	?	✓	✓	✓	?	✓	✓	~	✓	?	✓	✓	✓	?	✓	?	✓	?	?	?	✓	?	?	✓	~	?	?	✓	?	✓
**Adequate consideration of the relationship between researcher(s) and participants**	?	✗	✓	✗	✗	✓	✗	✗	✗	~	✗	✗	?	?	✓	✗	✗	✗	~	✗	✗	✗	✗	✗	✗	✗	✗	✗	~	?	✗	✗	✗	?	✗	✗	✗	✗	✗	✗	✗	✗	~
**Personal characteristics of the research team (COREQ)**	✓	✓	✓	✓	✓	✓	✓	✓	✓	✓	✓	✗	~	~	✓	~	✓	✗	~	✗	✗	✗	✗	✓	✗	~	✗	✗	~	~	✗	✗	~	~	✗	~	✗	~	✗	✓	✗	✗	~
Ethical issues considered	✓	✓	✓	✓	✓	✓	✓	~	✓	✓	~	✓	✓	~	✓	~	✓	~	~	~	~	~	~	✓	✓	✓	✓	?	✓	✓	✓	?	~	~	~	✓	~	✓	~	~	✓	~	~
**Sufficient and rigorous data analysis**	✓	✓	✓	✓	✓	✓	✓	✓	✓	✓	✓	✓	✓	✓	~	✓	~	✓	✓	✓	✓	✓	✓	~	✓	✓	✓	~	?	~	?	~	?	~	?	?	?	~	?	~	~	~	~
**Clear statement of findings**	✓	✓	✓	✓	✓	~	~	~	✓	~	~	✓	~	✓	~	✓	✓	✓	✓	✓	✓	✓	✓	✓	✓	~	✓	~	~	✓	✓	~	✓	✓	✓	✓	✓	✓	~	✓	~	✓	✓
Research valuability	✓	✓	✓	✓	✓	✓	✓	✓	~	✓	~	~	✓	✓	✓	✓	~	✓	✓	✓	✓	✓	✓	✓	✓	✓	✓	✓	✓	✓	✓	~	✓	✓	?	✓	✓	✓	~	~	~	~	✓

*✓* means ‘yes’, ✗ ‘no’,? ‘can’t tell’ and ~ ‘somewhat’. The numbering corresponds to the numbering used in the table presented in additional file 2. Table 1 Bold rows indicate the deciding criteria

The final sample of 43 studies comprised a total of 753 individuals, of whom 459 were male and 294 were female. Two lower-quality articles do not report exact gender distribution for their samples. The participants’ age ranged from 18 to 99 years, with mean and median between 40 and 79 years. Duration of illness varied from 3 months to 25 years. Given the common coexistence of heart diseases, most of the studies featured samples with multiple cardiac conditions. Most studies were conducted in Scandinavian countries, accounting for 18 of the 27 European studies. In Asia, six studies included participants from the Middle East, while one study each was conducted in Taiwan and Singapore. Additionally, six studies were based in the United States, and two in Canada. An overview of the sample characteristics is presented in Table 2. An additional overview of the included studies’ characteristics is provided in additional file 2.

**Table 2:** Sample characteristics

Characteristic	Total (%)	Characteristic	Total (%)
Male gender	459 (66)	Method of analysis	
Type of heart disease		Phenomenology	19 (44)
Cardiac Arrest	7 (16)	Content Analysis	16 (37)
Myocardial Infarction	11 (26)	Grounded Theory	8 (19)
Coronary Heart Disease	6 (14)	Text Condensation	1 (2)
Heart Failure	15 (35)	Geographical region	
Arrhythmia	4 (9)	Europe	27 (63)
Infective Endocarditis	1 (2)	Asia	8 (19)
Heart Valve Disease	2 (4)	North America	8 (19)

Our analysis identified four themes: (1) Feeling safe in my own body again, (2) Important relationships provide security and meaning, (3) Taking my life into my own hands again and (4) Living more consciously. In the following, each theme is presented in greater detail.

### Feeling safe in my own body again

“[The] hardest part to live with… [is] knowing it’s going to happen again.” [36] (p.232)

Patients experience the rupture of their normalcy through the loss of a sense of physical safety previously taken for granted, particularly in the case of an acute cardiac event. Regaining a sense of safety in their bodies is pivotal as it allows patients to gradually restore trust in their physical capabilities, thereby reclaiming a semblance of normalcy in their lives. Although this theme emerged as a significant concern across all heart disease types, it was particularly prominent in studies involving patients treated with medication or devices.

Initially, when patients face their illness for the first time and their sense of physical integrity is profoundly disrupted, medical monitoring and treatment, including surgeries, devices and medications, serve as sources of safety.

They are perceived as crucial supports and often likened to"life insurance,"a"second heart” or a “second doctor,” [37] (p.943) granting patients a sense of control and facilitating the restoration of bodily trust. Some patients even develop a personal bond with their device, referring to it as a “friend” [38] (p.519). Despite challenges such as dependency, side effects, or physical restrictions imposed by these interventions, patients recognize their benefits in terms of improved vitality and reduced fear of sudden death. A 68-year-old woman, who has lived with an implantable Cardioverter Defibrillator (ICD) for 10 years and experienced 3 electrical shocks and 2 generator changes, remembers:

“Before it was fitted, I was more ill. At that time, I said I was dying. I was worried (...) (nodding). Since it was fitted, those worries have gone. Since the battery was fitted, I've had fewer thoughts of death” [37] (p.939)

When the fear of sudden death has subsided and symptoms are less burdensome due to surgical procedures, devices, and medications, patients feel safe enough to explore their physical boundaries anew. Through the resumption of household chores or previous leisure activities, patients gradually acquire the ability to recognize and interpret physical signs.

“With time, I learned to recognize that pain, and learned a lot about my disease, why does it hurt, and how to help myself.” [39] (p.39)

In addition to the increased bodily awareness, knowing how to take good care of oneself fosters the patients’ confidence. Nevertheless, maintaining this safety requires ongoing effort. While some patients must motivate themselves to maintain a consistent exercise regimen, other patients face the challenge of reducing their activity levels, slowing down their daily routines, and adapting their environment to their new conditions to avoid overextending themselves. Especially in cases of heart disease with severe daily limitations, for instance, HF of III-IV New York Health Association (NYHA) class, or irreversible consequences following acute cardiac events, patients report feeling “imprisoned within [their] own body” [40] (p.7).

When patients succeed in adapting their environment to their new life circumstances and experience bodily safety, their bodies (at least partly) recede into the background, allowing them to enjoy life again.

“‘I realize that I cannot live like before, and I can only do things to the extent my chest pain permits! …And it was only a year after my heart attack that I realized that I had had a heart attack and had to take some medication and exercise until the end of my life, but it does not matter, I can still live, function, enjoy life…” 39 (p.37)

The climax of living, functioning, and enjoying life emerges as a fundamental experience among patients with heart disease, underscoring their journey from survival to enhancing physical capabilities and ultimately reclaiming a fulfilling life *within the boundaries of their illness*. For these patients, feeling safe in their bodies forms the foundation for the return to normalcy.

### Important relationships provide security and meaning

The importance of interpersonal relationships has emerged as another prominent theme. Positive relationship aspects were reported in nearly all studies, except in two studies on adjustment following first-time MI [23, 41], which focused primarily on self-image and identity; three studies on IHD [42–44]; and one study on HF [45], which exclusively presented negative relationship themes.

Facing their vulnerability, patients become acutely aware of the importance of interpersonal relationships, which offer a sense of security, meaning and a will to live. During the acute phase of the illness, connection to the healthcare system is crucial for the patients’ survival. However, when patients are discharged home after acute treatment, they often experience significant insecurity, as discussed in the previous theme. During this time, the presence of the family provides a crucial sense of security.

“I felt safe knowing that I wasn't alone, that I was with family” [22] (p.1237)

The reliable availability of others provides reassurance, both at hospital and at home. Receiving practical and emotional support from family and friends, significantly contributes to patients feeling secure and valued. Particularly CA and MI survivors describe that the awareness of their own mortality intensifies their relationships and leads to increased appreciation. That relationships impart a sense of meaning to one's life is particularly evident in studies on HF.

In addition to the support of family and friends, patients find regular contact with outpatient or rehabilitative care reassuring. Medical staff, through their reliable availability and empathetic, structured support, offer orientation, hope, and motivation.

“After a while I started to ask myself… ‘What can I do?’ ‘What can’t I do?’ sort of, and I had thousands of questions, so it was…it was great to go to see the nurse. …One did not really know what...well, how to…live, if…one would dare to do anything and so on, and…then” [40] (p.8)

In the uncertainty about what is possible for the body, suffering from severe HF, this patient turns to the nurse for professional assistance. Additionally, the exchange with other heart patients is described as helpful in addressing the question of how to live with a heart condition. Sharing information and hearing about others'experiences provides security and meaning, motivation and guidance for managing life with heart disease. One patient highlighted that gender-specific peer communication is important due to differing experiences between men and women coping with heart disease [46].

Although reliable relationships with others provide security, constant care also signifies a break from previous experiences. In their quest for stability and normality, patients strive to regain independence. Hence, half of the studies on CA, two on MI, three on HF, and one on IHD emphasize that it is important for patients to feel needed, making them feel like a"complete person"[37] (p.38). It stabilizes patients in two ways: First, being a role model for others or returning to work provides patients with a feeling of mattering to others, which enhances self-worth. Second, responsibility for others, especially for children, motivates patients to survive.

“I’ve got people who rely on me, so I can’t afford to die just yet … so that keeps me going. (Henry, 18/26)” [24] (p.1350)

While younger patients are more concerned about the care of their children, older patients worry about the emotional impact their death might have on others [47]. However, some patients feel relieved that they no longer must worry about others, particularly if their responsibility for others delayed their coming to terms with their own condition. For patients and their families, balancing between caring and granting autonomy is challenging but crucial to ‘live normally,’ as described by this 57-year-old woman:

“I have full support from my family, from my children. They advise me also; take care. But then you know, sometimes you live normal(ly), like normal people” [21] (p.395)

Relationships are an important element of a good life as they stabilize by providing security and meaning. Balanced relationships suggest a sense of normalcy.

### Taking my life into my own hands again

Just as it is important for patients in relationships to emancipate themselves from a passive, cared-for position over time, autonomy and self-determination are also significant in terms of shaping their lives. Although needs for autonomy and self-determination are described across all included types of heart disease, they appear to be particularly relevant for MI survivors and IHD patients. Three-quarters of IHD studies and all MI studies report on this issue.

Regaining mastery necessitates managing the dominance of the body, as it limits patients’ ability to conduct a self-determined life. Pushing the illness into the background serves as a coping strategy that patients employ to reclaim a sense of normalcy. This process can range from deliberately suppressing thoughts of illness to actively maintaining their previous physical appearance or striving to restore a former level of fitness to conceal the signs of the disease. The ability to continue activities that matter to them, despite limitations, is a crucial source of perceived normalcy and self-determination. Especially, maintaining an active lifestyle despite the disease holds special significance.

“Errmm…but I resented it and I would say I haven’t come to terms with the fact I can’t live my life the way I want to because I like to be doing things. I’m very fretty if I have nothing to do I like to be busy…errmm…I’m not interested in having half a life so even now there are days when I would push myself on a good day and I suffer on the bad day” [43] (p.268)

This patient equates a less active life due to chronic stable angina with living ‘half a life’. The terms ‘resenting the illness’ and ‘not coming to terms with it’ suggest that the patient rebels against the condition by continuing to pursue the individual interests. For some patients, in addition to engaging in active leisure activities, returning to work is an important part of an active lifestyle.

“‘When I work, I don't feel stress. I actually feel fulfilled and a great sense of achievement.” [48] (p.514)

For this 67-year-old man, returning to work despite his HF means experiencing fulfillment through achievement. Their occupation provides patients with feelings of being productive, needed and socially integrated, distracting them from their illness and giving the impression of normalcy. However, due to physical restrictions, not all patients can resume what is important to them with the same intensity as before the illness. Self-determination is then experienced when interests can still be pursued to some extent.

Living with CHD entails constant medical treatment. Therefore, patients also seek self-determination in their treatment. By providing feedback on medications and making autonomous treatment decisions, they strive to actively shape their care according to their individual standards and concerns. Consequently, patients value receiving sufficient medical knowledge to manage their condition. However, in some cases, self-determination includes the freedom to act contrary to medical advice, such as choosing their diet, engaging in travel or exercise without medical approval, or avoiding lengthy medical appointments.

“To accept the disease means to live in harmony with it, but again I say to some extent! Because I am not the type who will blindly adhere to some sort of ban.” [39] (p. 37)

In summary, this theme captures patients'efforts to maintain a sense of mastery over their lives by mitigating the disease's impact. When limitations cannot be overcome, patients negotiate the extent to which they can tolerate illness-related restrictions and find new ways to exercise self-determination within the constraints of the disease. A sense of self-determination is vital for living a good life despite CHD.

### Living more consciously

Half of the studies explicitly report that patients are grateful to be alive despite suffering from CA, MI, HF or IHD. Realizing life's fragility, patients develop deep gratitude toward healthcare providers for saving their lives and providing a second chance. While some patients adopt modesty and contentment with being alive, others emphasize the determination to"live each day to its fullest"

[44] (p.75) or to “follow [one’s] dreams” [20] (p.468). They become aware of what is important to them and shape their lives accordingly. This helps patients deal with the emotional challenges of SCHD, leading to perceived personal growth.

When life is threatened by disease, self-reflection and reassessment of priorities are motivated. Patients question their past behavior,"What was I doing wrong?"[39] (p. 38), and reflect on how to conduct their future lives,"What do I want to be remembered for?"[24] (p.1351). Defining their values and expectations for the future helps patients consciously lead a meaningful life.

For instance, patients recognize the importance of their health as a foundation for mobility and autonomy, making it a priority in lifestyle changes. However, despite finding meaning in investing in their health, some patients struggle with feeling"stuck in restrictions” [49] (p.370/1). In such cases, patients either report rebelling against the illness, as described in the previous section, or they experience self-determination by accepting their situation and regulating their emotions.

While 28 studies report that patients manage negative emotions by practicing patience, serenity and optimism, 13 studies with US American, European and Asian samples, report that patients’ faith helps them accept their disease and motivates them to cope with its challenges. Constructively engaging with and accepting their limitations without becoming discouraged is experienced by patients as personal strength, which may be mobilized by heart disease or arise because of it.

“I accepted the disease when I completely learned to live with it, when I realized that the disease was part of me, that I can handle, cope with it – my new normality! I have learned to recognize when I overdo it with my other life activities and have to stop for a bit. I need to take medication, I need to be careful but I can still live a satisfying life.” [39] (p.37)

For this patient, accepting the limitations after surviving MI forms the foundation for leading a satisfying life. Establishing a mindful, self-caring relationship with themselves enhances self-confidence and influences how patients interact with others. Some report learning to communicate their limits to others. Women, in particular, strive to reduce adherence to external expectations, while men report becoming more open about their feelings [49].

Shaping and living life consciously is an essential element of a good life catalyzed through heart disease. Patients develop a heightened appreciation of life, reassess their priorities, and adjust their lifestyles accordingly. Through negotiation with illness and mindful living, they find inner strength, and in some cases, experience personal growth.

## Interpretation and discussion

We conducted a meta-synthesis of 43 qualitative studies to expand the symptom-centered concept of HRQOL by identifying aspects of a good life as reported by patients with heart disease. To our knowledge, this is the first meta-synthesis to explore patients’ experiences through the lens of the philosophical concept of the Good Life, whereas previous meta-syntheses on heart disease have primarily focused on treatment aspects [16, 17, 50–53] and health behaviors [54–57]. However, few other meta-syntheses on QOL and self-care have emphasized the meaning of heart disease as biographical disruption and patients’ desire to return to normalcy [15, 58–60]. Strategies described for regaining a sense of normalcy include self-determination, finding meaning [57, 59, 60], reprioritizing values, pacing activities, listening to bodily signals [59], and living day-to-day to alleviate existential fears [60]. In line with those interpretations, our findings also reveal that heart patients strive to regain a sense of normalcy, which includes restoring trust in their vulnerable bodies, fostering balanced and meaningful relationships, and actively shaping their lives. Confronting their vulnerability fosters a renewed appreciation for life, encouraging patients to live more consciously and prioritize core values. Despite these broad similarities, our findings also highlight the need to feel useful and the influence of age on relationship dynamics.

Moreover, our study differs methodologically by including various cardiac conditions, which allowed us to observe differences in the relative importance of components of a good life across these conditions. This approach highlighted, for instance, the particular importance of self-determination in studies on MI and IHD and revealed varying themes of relationship conflict across patient groups. Unlike prior reviews that primarily applied meta-ethnographic [16, 58, 60] or thematic analysis [15, 17, 50, 60], we used IPA to synthesize primary research data. The combination of phenomenological and hermeneutic techniques enabled us to develop categories that capture the core structure of patients’ ideas of a good life. These categories form the basis for our proposed conceptualization of QOL, which aligns more closely with the WHO definition of QOL than with the HRQOL framework.

### The physical dimension – bodily trust

We argue that physical safety is fundamental to heart patients, allowing them to rebuild a sense of normalcy. This aligns with Maslow's hierarchy, where lack of bodily safety can lead to illness or hinder recovery [61]. According to phenomenology, in illness, the body (*Körper*) no longer aligns with the lived body (*Leib*), leading to heightened awareness of physical limitations. This dissonance, as noted by Merleau-Ponty, results in an intensified focus on the body, limiting patients’ experiences and actions [62]. The desire for security reflects an attempt to reconcile *Körper* and *Leib*. Achieving a minimum level of health is essential for fostering autonomy and connectedness, both crucial components of a good life.

### The social dimension – security and meaning through relationships

Supportive and meaningful relationships are vital for shaping a good life with SCHD, offering security akin to medical devices. However, patients often feel dependent on others while simultaneously experiencing isolation [62, 63]. Feelings of otherness and existential loneliness frequently emerge in chronic illness contexts [62, 63], particularly when confronting mortality [64]. Grief, despair, and fear become palpable when patients feel fundamentally isolated due to a lack of understanding from others [64]. Our findings support existing literature that suggests peer support offers a vital sense of being understood [65].

On the other hand, balancing relationships through caregiving, work, or mentorship fulfills patients' needs for mattering and maintains self-esteem, positively correlating with happiness, purpose, and well-being [66]. Erikson’s concept of generativity aligns with our findings that helping others can aid patients in coping with mortality, although this theme remains underexplored in heart disease [67].

### The psychological dimension – self-determined and conscious living

Autonomy and self-determination are crucial for a fulfilling life. Chronic illness often involves losses, and losing agency can be particularly distressing [25, 62]. Our results reflect a patient-driven effort to regain autonomy as a form of resistance to illness. Meaningful activities, such as work and hobbies, reinforce identity and purpose; however, efforts to assert independence can sometimes lead to non-adherence to treatment. Integrating self-determination into clinical care could enhance adherence by supporting autonomy.

Living more consciously, on the other hand, reflects acceptance of limitations. Patients demonstrate emotional discipline by focusing on their remaining abilities and cultivating optimism [62], contributing to mastery and self-efficacy associated with lower cardiovascular mortality [68]. However, chronic illness trajectories are rarely linear, with the processes of observing bodily reactions, adapting to limitations, and achieving accomplishments remaining ongoing [69].

### Temporality – a forgotten element in HRQOL research

Our analysis shows that heart disease impacts patients'experience of time. As discussed in the first part of our meta-synthesis, many perceive it as a rupture in their life trajectory, rendering the future uncertain and disconnecting them from their past due to illness-related limitations. In response, some patients focus on the present, while others experience a loss of meaning along with the loss of a secure future. Medical devices, viewed as life insurance, impose their own rhythms, requiring regular charging or replacement. Physical limitations and lifestyle changes may necessitate the pacing of daily activities, leading to feelings of premature ageing, while patients’ actual age further influences caregiving dynamics.

Several studies indicate that the experience of time is altered in somatic conditions like cancer [70, 71] as well as in psychiatric conditions such as depression [72], psychotic disorders [73], and personality disorders [74]. However, the direction of this relationship remains unclear, as does the question of whether a fundamentally altered experience of time due to severe or chronic somatic disease might itself be a causal factor in comorbid mental disorders.

### Limitations

Our study has several limitations: First, the types of severe or chronic heart disease included were not defined a priori. Instead, we searched broadly for publications on heart disease and made inclusion decisions based on the descriptions of the specific condition and participants’ accounts of perceived life disruptions. Second, it remains unclear how comorbidities or aging influence patients’ perceptions of a good life. Missing data on age, gender, illness duration and severity limited the idiographic elements typical in IPA. While our primary aim – to identify common aspects of a good life with heart disease – was achieved, the full diversity of the phenomenon could not be captured. Additionally, the sample is biased toward western, educated, industrialized, rich, and democratic (WEIRD) regions, with no studies from Latin America, Africa, or Australia.

## Conclusion

Our meta-synthesis identified core elements that patients with SCHD associate with a good life. Existing HRQOL measures, however, only partially capture patients’ perspective; they primarily assess physical and psychological symptom burden and its impact on abilities and social well-being, rather than addressing patients'experiences of bodily safety, connectedness, mattering, meaning, and self-determination. To better reflect patients’ lived experiences, we recommend expanding HRQOL measures to include these fundamental aspects. Furthermore, it is essential to consider whether patients recognize their priorities and align their lives with these values, as this provides meaning and purpose. To support patients in pursuing a fulfilling life, healthcare providers should adopt a life-course perspective, fostering coherence between past, present, and future while instilling hope.

## Supplementary Information

Additional file 1.

Additional file 2.

## Data Availability

Data is provided within the manuscript or supplementary information files.

## Abbreviations

QOL: Quality of Life
WHO: World Health Organization
HRQOL: Health-Related Quality of Life
MI: Myocardial Infarction
CA: Cardiac Arrest
HF: Heart Failure
IHD: Ischemic Heart Disease
SCHD: Severe or Chronic Heart Disease(s)
AF: Atrial Fibrillation
PTG: Posttraumatic Growth
IPA: Interpretative Phenomenological Analysis
ENTREQ: Enhancing Transparency in Reporting the Synthesis of Qualitative Research
PiCos: Population, Investigated condition, Context of interest, and type of Studies included
MeSH: Medical Subject Headings
PRISMA: Preferred Reporting Items for Systematic Reviews and Meta-Analyses
CASP: Critical Appraisal Skills Program
COREQ: Consolidated Criteria for Reporting Qualitative Research
ICD: Implantable Cardioverter Defibrillator
NYHA: New York Health Association
