# Supplementary material for: Working towards a new normal: a meta-synthesis of patient-reported aspects of a good life with heart disease
Source: Health Qual Life Outcomes. 2025 Jun 4;23:56. doi: 10.1186/s12955-025-02388-6 (PMC12135301; doi:10.1186/s12955-025-02388-6)
Supplement: Supplementary file 1 — Additional file 1. [file 12955_2025_2388_MOESM1_ESM.docx]

**Table 1.** Search strings used in PubMed, all applied to titles and abstracts

|  | **Population** | **Methodology** | **Phenomenon** |
| --- | --- | --- | --- |
| **Operators** | **AND** | **AND** | **AND** |
|  | **Heart Diseases**  **(MeSH)** | **Qualitative Research**  **(MeSH)** | **Quality of Life (MeSH)** |
| **OR** |  | Semi-Structured Interview* | **Psychological Adaptation (MeSH)** |
| **OR** |  | Unstructured Interview* | Good Life |
| **OR** |  | In-depth Interview* |  |
| **OR** |  | Foc* Group* |  |
| **OR** |  | Phenomenol* |  |
| **OR** |  | Grounded Theory |  |
| **OR** |  | Anthropol* |  |
| **OR** |  | Ethnograph* |  |
| **NOT** | **Congenital Heart Disease (MeSH)** |  |  |

**Table 2.** Search strings used in PsycInfo, PsycArticles and PSYNDEX, all applied to titles and abstracts

|  | **Population** | **Methodology** | **Phenomenon** |
| --- | --- | --- | --- |
| **Operators** | **AND** | **AND** | **AND** |
|  | **Heart Diseases (I)** | **Qualitative Methods (I)** | **Quality of Life (I)** |
| **OR** | Angina Pectoris | Thematic Analysis | (Spiritual/Subj.) Well Being |
| **OR** | Coronary Artery Disease | Interviews | HRQol |
| **OR** | Coronary Thromboses | Self-Report | Life Style (Changes) |
| **OR** | Heart Arrythmias | Focus Group (Interview) | Life Changes |
| **OR** | Bradycardia | Phenomenology | Life Satisfaction |
| **OR** | Heart Fibrillation | Grounded Theory | Good Life |
| **OR** | Tachycardia | Narrative Analysis | **Emotional Adjustment (I)** |
| **OR** | Myocardial Infarctions | IPA |  |
| **OR** | CPR |  |  |
| **NOT** | Congenital Heart Disease |  |  |
